# Super Field-of-View Lensless Camera by Coded Image Sensors †

**DOI:** 10.3390/s19061329

**Published:** 2019-03-16

**Authors:** Tomoya Nakamura, Keiichiro Kagawa, Shiho Torashima, Masahiro Yamaguchi

**Affiliations:** 1 School of Engineering, Tokyo Institute of Technology, Kanagawa 226-8502, Japan; torashima.s.aa@m.titech.ac.jp (S.T.); yamaguchi.m.aa@m.titech.ac.jp (M.Y.); 2 PRESTO, Japan Science and Technology Agency, Saitama 332-0012, Japan; 3 Research Institute of Electronics, Shizuoka University, Shizuoka 432-8011, Japan; kagawa@idl.rie.shizuoka.ac.jp

**Keywords:** computational imaging, lensless camera, CMOS image sensor, compressive sensing

## Abstract

A lensless camera is an ultra-thin computational-imaging system. Existing lensless cameras are based on the axial arrangement of an image sensor and a coding mask, and therefore, the back side of the image sensor cannot be captured. In this paper, we propose a lensless camera with a novel design that can capture the front and back sides simultaneously. The proposed camera is composed of multiple coded image sensors, which are complementary-metal-oxide-semiconductor (CMOS) image sensors in which air holes are randomly made at some pixels by drilling processing. When the sensors are placed facing each other, the object-side sensor works as a coding mask and the other works as a sparsified image sensor. The captured image is a sparse coded image, which can be decoded computationally by using compressive sensing-based image reconstruction. We verified the feasibility of the proposed lensless camera by simulations and experiments. The proposed thin lensless camera realized super-field-of-view imaging without lenses or coding masks and therefore can be used for rich information sensing in confined spaces. This work also suggests a new direction in the design of CMOS image sensors in the era of computational imaging.

## 1. Introduction

Computational imaging is an imaging method based on a combination of optical encoding and computational decoding [1]. A computational lensless camera is a lensless camera that works on the basis of computational imaging instead of lens-based optical imaging [2]. The lensless imaging frees the camera from the need of optical focusing. This allows the camera to be implemented with ultra-thin, miniature hardware [3]. In a lensless camera, a lens system is typically replaced with a coded aperture such as a coding mask, which makes the inverse problem numerically invertible. As the coded aperture, amplitude masks [4,5,6] or phase-modulation optics [7,8,9,10] have been installed in front of the image sensor. In computational lensless imaging, the spatial information of a subject is coded by the coded aperture, and the optically-coded image is sampled by the image sensor. After exposure, the captured coded image is numerically decoded into the original image of the subject. In the decoding, the inverse problem of coded sensing is solved by signal processing.

**Figure 1:**
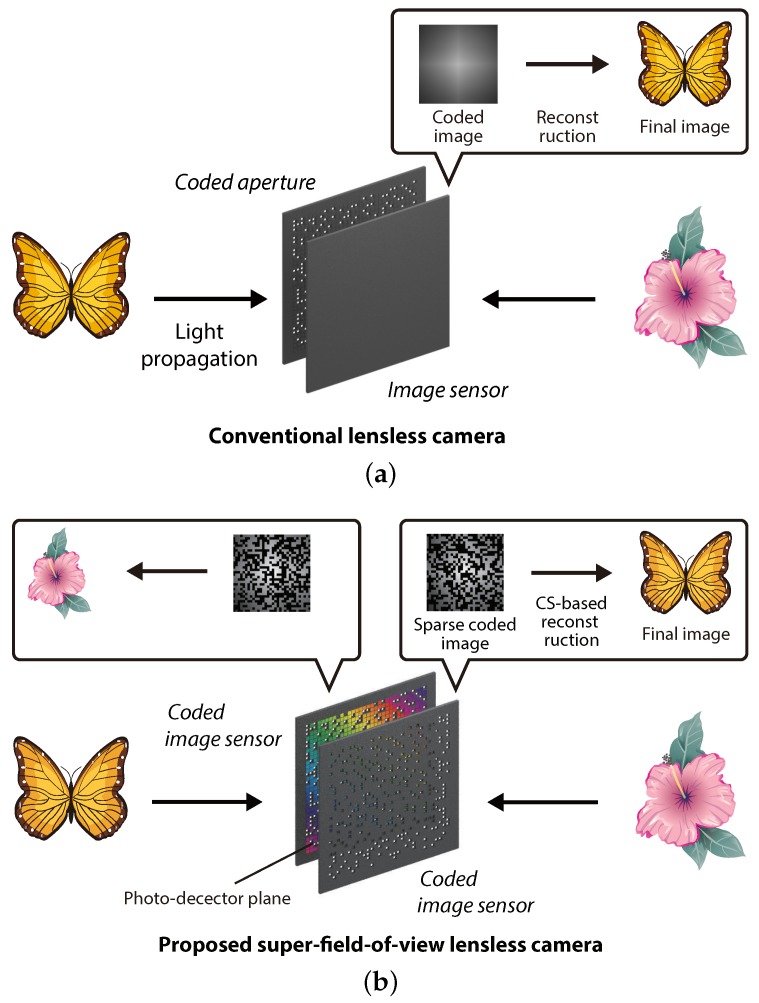
Concept of (**a**) a conventional lensless camera and (**b**) the proposed super-field-of-view lensless camera using coded image sensors (CISs). The photo-detector plane faces inside the optical system in both figures.

Existing architectures of lensless cameras are based on the axial arrangement of an image sensor and a coded aperture, like that shown in Figure 1a. This means that the back side of the sensor cannot be captured. In other words, the field-of-view (FOV) in mask-based lensless imaging is limited to 180 degrees in the forward direction only. To extend the limit, the use of multiple lensless cameras is the most straightforward approach; however, this is naturally at the sacrifice of the simplicity and thinness of the system because additional lensless cameras are required. In previous studies, methods to utilize a multiple-scattering medium as an optical encoder have been proposed to extend the FOV in a single-sensor lensless camera [8,11]. Multiple scattering can effectively enhance the FOV as indicated in the papers; however, the backside of the image sensor cannot be captured in the methods. For example, the work in Ref. [8] reported a 180-degree FOV lensless camera; however, the image shooting of the rear space from the image sensors is impossible in principle. In another previous study, a wide-FOV lensless camera by multiple curved and flat mirrors was also proposed for the purpose of wearable imaging [12]. This method can also extend the FOV; however, the optical design involving four mirrors still needs thickness in optical hardware because axial and lateral intervals are required between mirrors and an image sensor.

In this paper, we propose a lensless camera with a novel design that is capable of capturing images of the front and back sides at the same time. Figure 1b shows the concept of the proposed super-FOV lensless camera. The proposed camera is composed of an axial arrangement of two coded image sensors (CISs), which are complementary-metal-oxide-semiconductor (CMOS) image sensors in which air holes are randomly formed at some pixels by drilling processing. In this arrangement, the photo-detector planes of the sensors face each other. Thanks to the drilling-based physical sparsification of the image sensor, the sensor works not only as an image sensor, but also as a coded aperture for the opposing image sensor. After image capturing, the captured sparse coded images are decoded into the original images of the subjects by a numerical image-reconstruction algorithm based on the compressive sensing (CS) framework [13,14,15]. Section 2 describes the methodology of the proposed lensless imaging. Section 3 shows the concept verification and performance analysis by numerical simulations. Section 4 shows the experimental verification using a real mask-based lensless camera and simulated air holes. This paper is an extended version of our earlier conference proceeding on this camera system [16]. The work in Ref. [16] reported the concept and preliminary results briefly. This paper presents the detailed methodology and comprehensive performance analysis by extended simulations and experiments.

## 2. Methods

**Figure 2:**
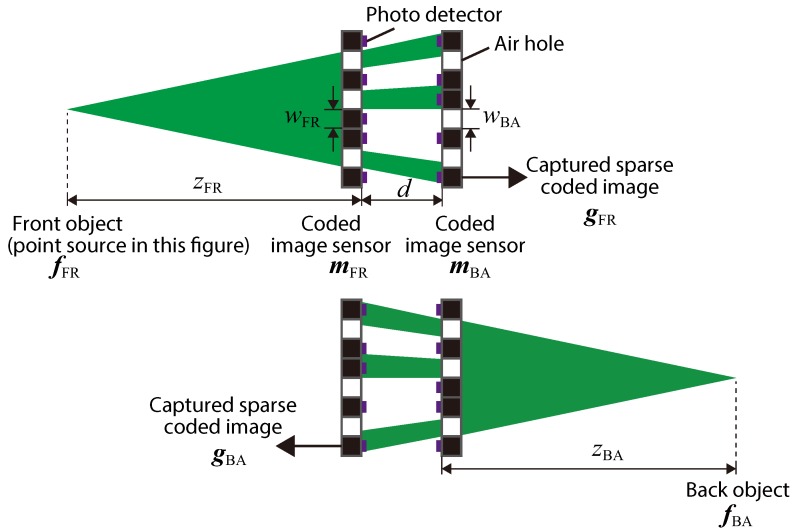
Parameter definition for the proposed camera. The illustration of each image sensor represents the amplitude transmittance of the pixel area, where white pixels correspond to air holes. The green area represents the light transmittance in an optical system when the object is a point source.

Figure 2 presents a schematic view of the optical hardware of the proposed camera, with definitions of the system parameters. As the optical hardware, two CISs that included both photo-detectors and air holes were provided. The photo-detector planes faced the inside of the optical system. We assumed that the air holes in the image sensor were implemented by drilling processing, where the drilled pixels were chosen randomly. In the setup, we called the object at the left side in figure the front object $f_{\mathrm{FR}}\in R^{N_{f}\times 1}$ and the object at the right side the back object $f_{\mathrm{BA}}\in R^{N_{f}\times 1}$. $N_{f}$ is the pixel count of an object assumed in imaging, and $R^{N_{f}\times 1}$ presents a real matrix with $N_{f}\times 1$ elements. Here, each vector represents the light intensity of an object. Similarly, we denoted the front CIS as $m_{\mathrm{FR}}\in R^{N_{f}\times 1}$ and the back CIS as $m_{\mathrm{BA}}\in R^{N_{f}\times 1}$. Note that the values of all the elements in a sensor vector were zero or one, which physically express the amplitude transmittance of the pixels. In addition, we also denoted the intensity vectors of captured sparse coded images as $g_{\mathrm{BA}}\in R^{N_{g}\times 1}$ and $g_{\mathrm{FR}}\in R^{N_{g}\times 1}$, respectively. $N_{g}$ is the pixel count of a captured image. The sizes of the square air holes in each sensor are denoted as $w_{\mathrm{FR}}$ and $w_{\mathrm{BA}}$. The axial interval between the two sensors is denoted as *d*, and the distances between the sensors and the objects are denoted as $z_{\mathrm{FR}}$ and $z_{\mathrm{BA}}$, respectively.

The forward model of the coded image sensing by the proposed camera is as follows:

(1) $$\begin{array}{cc} g_{\mathrm{FR}} & =M_{\mathrm{BA}}\left(h_{\mathrm{FR}}*f_{\mathrm{FR}}\right), \end{array}$$

(2) $$\begin{array}{cc} g_{\mathrm{BA}} & =M_{\mathrm{FR}}\left(h_{\mathrm{BA}}*f_{\mathrm{BA}}\right), \end{array}$$

where $M_{\mathrm{BA}}\in R^{N_{g}\times N_{f}}$ and $M_{\mathrm{FR}}\in R^{N_{g}\times N_{f}}$ are the matrices expressing the sparse sampling by CISs whose structures are $m_{\mathrm{BA}}$ and $m_{\mathrm{FR}}$; and $h_{\mathrm{FR}}\in R^{N_{f}\times 1}$ and $h_{\mathrm{BA}}\in R^{N_{f}\times 1}$ are the vectors of optical point-spread functions (PSFs), which are the optical shadows of the opposing CISs. The optical PSFs are modeled as follows:

(3) $$\begin{array}{cc} h_{\mathrm{FR}} & =W_{\mathrm{zFR}}m_{\mathrm{FR}}*b, \end{array}$$

(4) $$\begin{array}{cc} h_{\mathrm{BA}} & =W_{\mathrm{zBA}}m_{\mathrm{BA}}*b, \end{array}$$

where $W_{\mathrm{zFR}}\in R^{N_{f}\times N_{f}}$ and $W_{\mathrm{zBA}}\in R^{N_{f}\times N_{f}}$ are the matrices expressing the scaling of geometrical shadows depending on the distance to the object and $b\in R^{N_{f}\times 1}$ is the blur kernel of diffraction caused by the light propagation between the two image sensors. Here, * denotes convolution. We assume that the optical PSF is approximately space-invariant [10].

In imaging, the patterns of the optical PSF h and the sparse sampling W can be determined by calibration, and g can be obtained by image capturing. Basically, the intensity image of the original object f can be numerically reconstructed by solving the inverse problem of the forward model in Equations (1) and (2). However, the inverse problems are ill-posed because of the sparse sampling by the CISs. In other words, $N_{g}<N_{f}$ in the inverse problem of the proposed camera. Therefore, we adopt the CS framework [13,14] for image decoding [15]. In the CS framework, the original image can be reconstructed uniquely even when $N_{g}<N_{f}$ by solving the following problem:

(5) $$\begin{array}{cc} {\hat{f}}_{\mathrm{FR}} & =\underset{f_{\mathrm{FR}}}{\mathrm{argmin}}\left|\right|g_{\mathrm{FR}}-M_{\mathrm{BA}}\left(h_{\mathrm{FR}}*f_{\mathrm{FR}}\right){\left|\right|}_{\ell_{2}}+\tau ||\Phi \left(f_{\mathrm{FR}}\right){\left|\right|}_{\ell_{1}}, \end{array}$$

(6) $$\begin{array}{cc} {\hat{f}}_{\mathrm{BA}} & =\underset{f_{\mathrm{BA}}}{\mathrm{argmin}}\left|\right|g_{\mathrm{BA}}-M_{\mathrm{FR}}\left(h_{\mathrm{BA}}*f_{\mathrm{BA}}\right){\left|\right|}_{\ell_{2}}+\tau ||\Phi \left(f_{\mathrm{BA}}\right){\left|\right|}_{\ell_{1}}, \end{array}$$

where $\left|\right|\cdot {\left|\right|}_{\ell_{2}}$ and $\left|\right|\cdot {\left|\right|}_{\ell_{1}}$ denote the $\ell_{2}$-norm and $\ell_{1}$-norm of a vector and τ is a constant. Φ(·) is a regularizer, which corresponds to a linear transformation for sparse modeling, such as the discrete wavelet transform, the discrete cosine transform, and so on. In this paper, we specifically used the two-dimensional (2D) total variation (TV) [17] as the regularizer, which is computed as follows:

(7) $$\begin{array}{c} \left|\right|\Phi_{\mathrm{TV}}{\left(f\right)||}_{\ell_{1}}=\sum_{n_{x}}\sum_{n_{y}}\left|\nabla f_{x,y}\right|, \end{array}$$

where $\nabla f_{x,y}$ is the differential images of f along the horizontal and vertical directions and $n_{x}$ and $n_{y}$ are the pixel counts along the two axes of the 2D image. To implement the well-conditioned inverse problem, the pattern of drilling on image sensors was designed as a random pattern [18].

**Figure 3:**
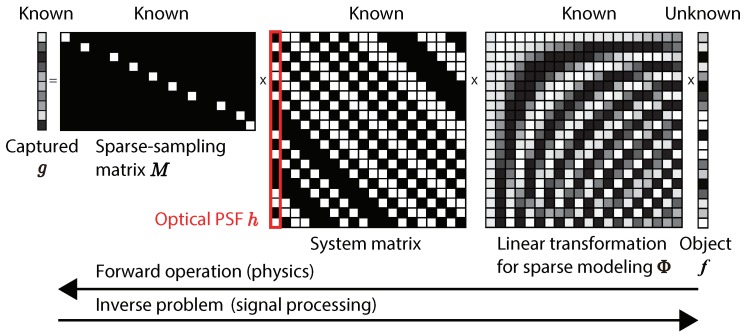
Matrix-vector expression of the imaging model with a one-dimensional (1D) object.

For a more intuitive understanding, the matrix-vector expression of the 1D imaging model in the proposed method is shown in Figure 3. As mentioned above, we assumed that the object after a linear transformation $\Phi (f_{\mathrm{FR}})$ was sparse. Since the optical image on the image plane was the convolution of the mask and the object, the optical system matrix was the Toeplitz matrix whose column represents the optical PSF. By the sampling $M_{\mathrm{BA}}$ of a CIS, the length of the captured coded image was made shorter than that of the object. Therefore, the inverse problem of the linear forward imaging model is ill-posed. To solve this, a technique based on CS is necessary.

In practice, the blur kernel b can be directly measured; however, it can also be analyzed by a diffraction calculation [19]. The 1D diameter of the kernel $b_{w}$ can be roughly estimated by using a simple model based on the Fraunhofer approximation as follows:

(8) $$\begin{array}{c} b_{w}\approx \frac{\lambda d}{w}, \end{array}$$

where λ is the wavelength and *w* is the width of an air hole. In this paper, we denote the diameter $b_{w}$ by the distance between first-zero values in the diffraction pattern expressed by the sinc function. Considering diffraction, the size of the optical shadow of the air hole on the image plane is simply formulated as follows:

(9) $$\begin{array}{c} w^{\prime }=w+b_{w}. \end{array}$$

**Figure 4:**
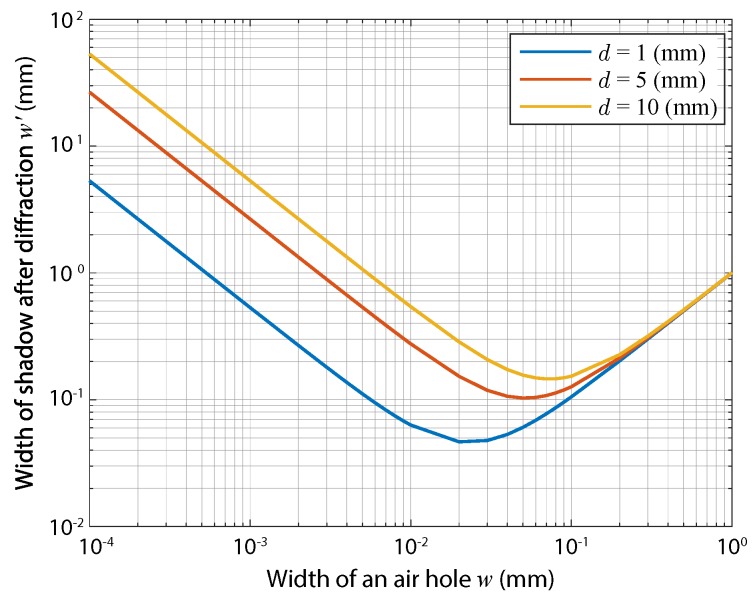
The size of the optical shadow of a small air hole in a CIS, which is formed on another CIS after diffraction. The color of the lines expresses the axial distance between the two image sensors, ranging from 1 mm–10 mm.

Example values of $w^{\prime }$ at a wavelength of 532 nm are plotted in Figure 4. As shown in the figure, there are optimal sizes of the air hole to give the smallest image-spot size after diffraction. The size of the optical shadow of a hole directly corresponds to the highest spatial optical resolution in aperture-shadow-based lensless imaging. Note that the spatial resolution of the lensless camera, independently of the optical resolution, is also restricted by the spatial sampling resolution, which is physically determined by the pixel pitch.

## 3. Simulations with Numerical Data

We performed simulations with numerically-generated data to validate the proposed imaging model and its performance. In this paper, we assumed an optical system like that in Figure 2 and only simulated 1D imaging, as shown in the upper illustration, for simplicity. Note that the same discussion can be adopted for imaging in another direction, as shown in the lower illustration of Figure 2. For an explanation, here we refer to the captured object as the front object and the object-side image sensor as the front sensor. In this simulation, we generated captured data by emulating imaging and decoded it via a reconstruction algorithm.

**Figure 5:**
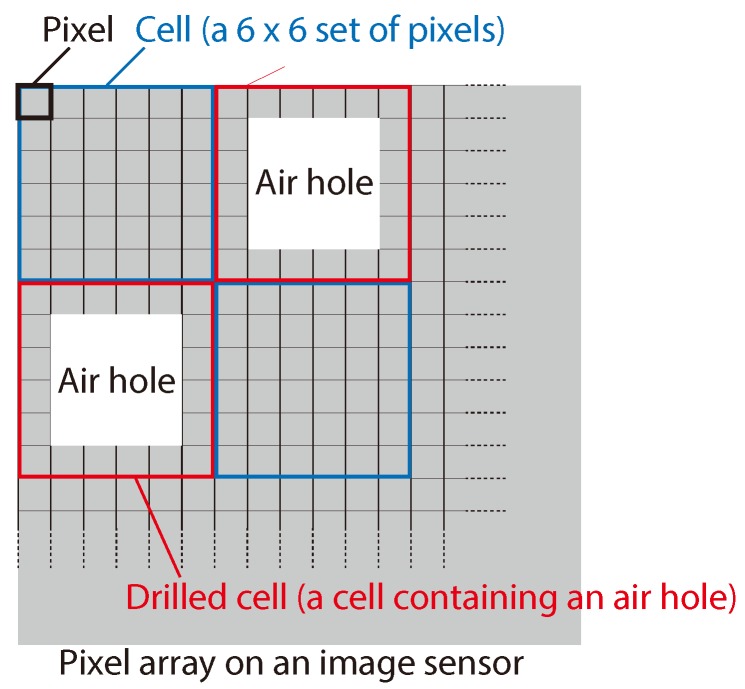
Definition of *cell* and its relation with air holes in a pixel array on an image sensor.

Here, we define the new term *cell* in a pixel array as a set of 6×6 pixels, as shown in Figure 5. We randomly classified the cells into normal cells and drilled cells, where each drilled cell contained an air hole at the center, as in the figure. For the discussion in this paper, we defined the term air-hole ratio as the ratio of drilled cells to normal cells. The parameters of the optical system, including the image sensors, are summarized in Table 1. With these parameters, the image sensor can be regarded as an array of 64×64 cells, whereas the total pixel count in the image sensor was 384×384.

As the procedure for the simulation, first, the coded image formed on the back sensor was numerically generated by the convolution of the subject and the optical PSF. The optical PSF was calculated by the convolution of the front sensor and the diffraction-based blur kernel. Note that here, we modeled the blur kernel (b in Equation (3)) as a 2D Gaussian profile whose full-width at half-maximum (FWHM) was determined by that in the Fraunhofer-diffraction pattern of a small air hole ($b_{w}$ in Equation (8)). Afterward, the digital sampling of the coded image formed by a CIS was emulated with image sparsification, multiplying by the photon-shot noise, and 12-bit quantification. Finally, the captured data were decoded into an original image of the subject using a CS-based image-reconstruction algorithm. For the image reconstruction, we used the TwIST algorithm [20], which iteratively solves Equation (5).

**Figure 6:**
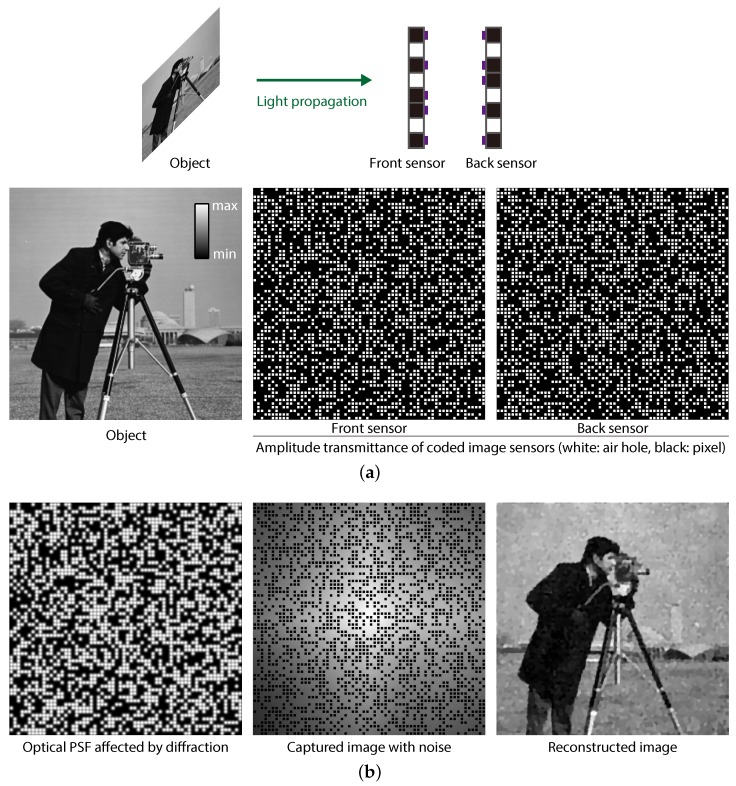
(**a**) Setup for imaging simulation including a subject and the amplitude transmittance of two CISs when the air-hole ratio was 50%. (**b**) Results of the simulation including the optical point-spread function (PSF), a captured sparsely-sampled coded image with noise, and the reconstructed image.

The subject and the amplitude transmission of both the front and back sensors used for the simulation are shown in Figure 6a, together with an illustration of the assumed optical setup. In the figures of the image sensors, a white pixel physically means an air hole (drilled), whereas a black one indicates an opaque pixel (not drilled). In this simulation, we set the air-hole ratio to 50% for both sensors, which is an example parameter. We designed the back sensor by rotating the front sensor by 90 degrees, which enabled differentiation of structures of two CISs manufactured with the same air-hole structure. As the subject, we used the standard image “Cameraman”.

**Figure 7:**
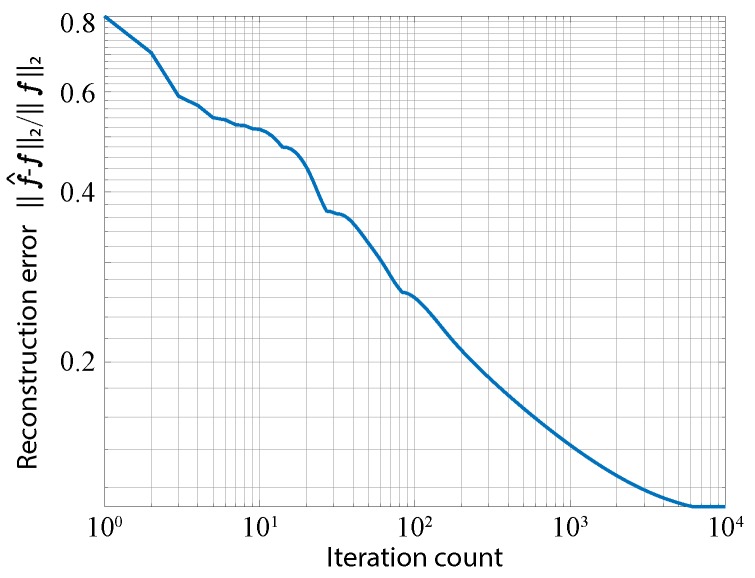
Convergence of reconstruction error with iteration in image decoding.

The simulation results are shown in Figure 6b. The optical PSF was a blurred image of the amplitude transmittance of the front sensor. To emulate photon-shot noise, the sparse-coded image was multiplied by Poisson noise, where the simulated mean signal-to-noise ratio (SNR) was 51.6 dB. As expected, the appearance of the captured image was not similar to the original object; nevertheless, the image information was encoded inside the image, though it was sparsified. The reconstructed image is shown in the right column of Figure 6b. For image decoding, τ in Equation (5) was adjusted to $1\times 10^{-6}$ experimentally. The number of iterations of the algorithm was set to 10,000 to ensure convergence of the cost function in optimization. Figure 7 shows the plot of converging reconstruction error with iterations. This indicates that the iteration count used in this simulation was enough for the convergence of optimization. As a result, we confirmed that an image of the object was successfully reconstructed. The peak SNR (PSNR) of the reconstructed image was 24.7 dB. The total processing time was 3.82 min when running MATLAB R2017b on a computer with an Intel Xeon E5-2697 v4 CPU, an NVIDIA GeForce GTX 1080Ti, and 128 GB RAM.

**Figure 8:**
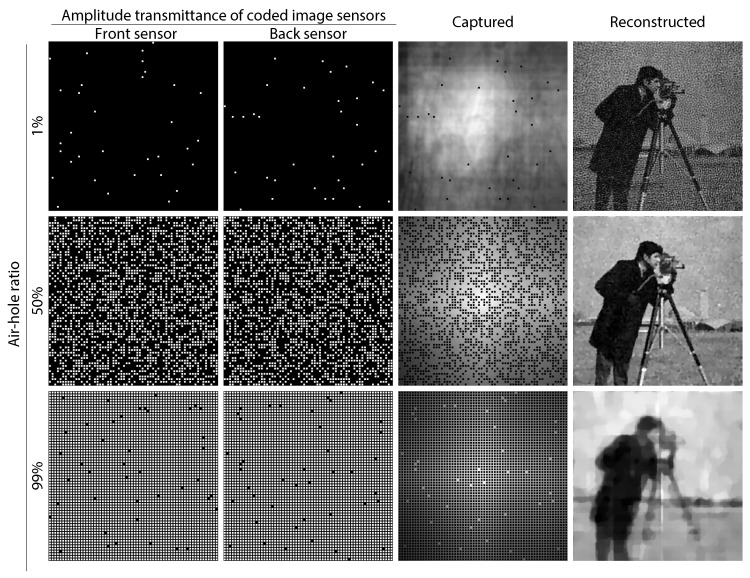
Simulation results obtained with different air-hole ratios of the CISs. Used sensors, captured images, and reconstructed images when the air-hole ratio was 1%, 50%, and 99%.

**Figure 9:**
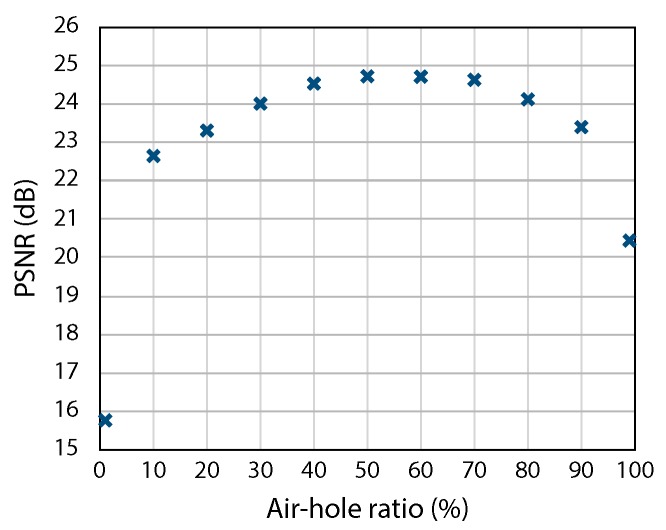
PSNRs of the reconstructed images in simulations, where the air-hole ratio ranged from 1%–99%.

We also investigated the effect of the air-hole ratio in the image sensor. Here, we assumed that the front and back image sensors were implemented with the same air-hole ratio. In such a case, there is a trade-off between the amount of transmissive light at the front sensor and the sampling count at the back sensor. Increasing the air-hole ratio resulted in a better SNR due to the higher detected photon count; however, it simultaneously resulted in reduced fidelity of the CS-based image reconstruction due to the sparser sensing. Therefore, the air-hole ratio should be designed optimally by considering the imaging situation, such as the environmental light intensity, the object’s sparseness, and so on. Figure 8 shows examples of the reconstructed images obtained with different air-hole ratios. In the figure, the air-hole ratio was set to 1%, 50%, and 99% from the left to the right. In this simulation, the change of the ratio directly affected the amount of photon-shot noise and the sampling count of the captured data. Note that the standard deviation of the photon-shot noise was equal to the square root of the detected photon count. Looking at the visual appearance in the figure, a lower ratio resulted in noisy image reconstruction, whereas a higher ratio resulted in lower-fidelity reconstruction, as expected. Figure 9 shows the PSNRs of the reconstructed images obtained when changing the ratio from 1%–99%. For example, the PSNR with ratios of 1%, 50%, and 99% were 15.8 dB, 24.7 dB, and 20.4 dB, respectively. The results quantitatively indicated that an air-hole ratio of around 50% resulted in the best final image quality under the example conditions used in the simulation.

## 4. Experiments Using a Mask-Based Lensless Camera and Simulated Air Holes

**Figure 10:**
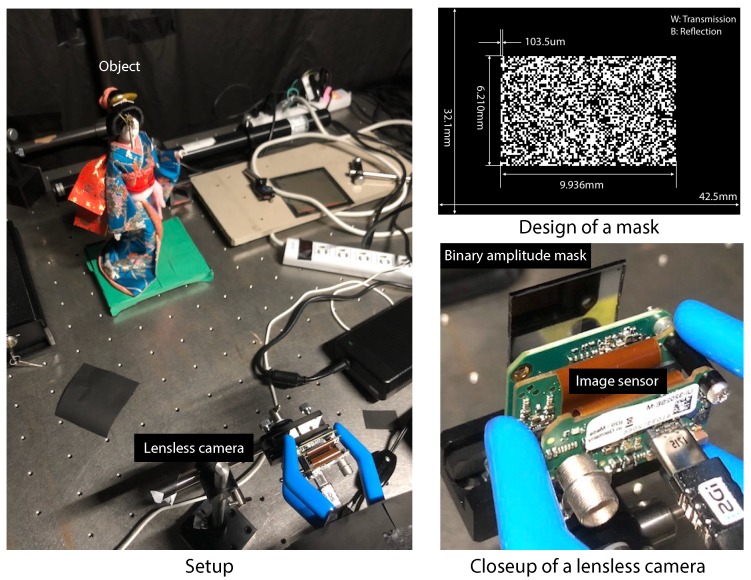
Experimental setup for capturing coded images using a mask-based lensless camera. The mask pattern indicates its amplitude transmission.

We further investigated the validity of the proposed imaging method using data experimentally captured by a real mask-based lensless camera. Figure 10 shows the experimental setup. To implement a lensless camera, first, we designed and fabricated a binary amplitude mask with a randomly-coded structure. The mask was implemented by chromium deposition on a synthetic-silica plate. It was placed in front of a color CMOS image sensor (UI-3202SE-C by IDS), which contained 4104×3006 pixels with a 3.45 μm pitch. Note that we resized the image to 512×512 pixels after the image capturing and demosaicing to accelerate the CS-based image decoding. The combination of the image sensor and the mask worked as the optical hardware of a lensless camera. As the subject, we placed a liquid-crystal display (LCD) or a doll in front of the lensless camera. In this experiment, we set the distance between a subject and a mask as 40 cm. The PSF of the lensless camera was calibrated experimentally by capturing the image of a point light source. When imaging, the space between the mask and the sensor was covered by light-shielding tape. In this experiment, random sparse sampling was computationally emulated. We will fabricate a real CIS in future work. For color-image acquisition, we performed image decoding for each color channel independently, where the decoding method for each channel was the same as that in the previous section. For simplicity, we assumed that the air holes were implemented pixel-by-pixel randomly.

**Figure 11:**
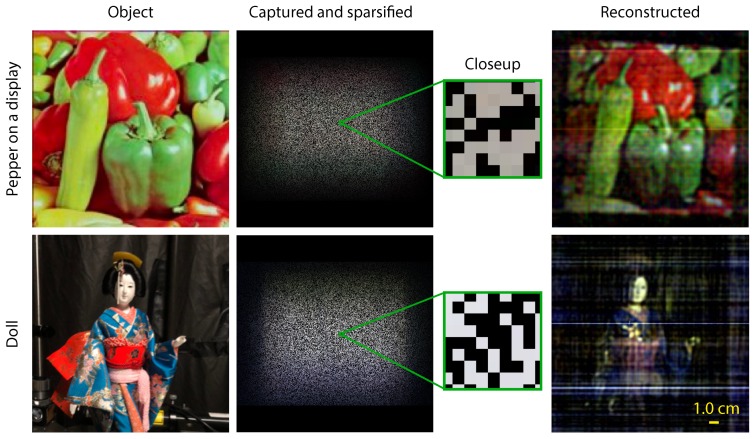
Experimental results using a real mask-based lensless camera and simulated air holes. The results includes subjects, captured and sparsified images, and reconstructed images. The scale bar indicates 1.0 cm.

Figure 11 shows the results of the experiment. As the subjects, we used a “Pepper” image displayed on an LCD and a Japanese doll as a diffuse object. In the experiment, the coded image was captured just by exposure of an image sensor, where image encoding was accomplished optically. Unlike simulations, noise was not added computationally because it was generated by the physical phenomena of devices. After capturing the raw coded image, image demosaicing was performed for generating a color coded image, and 50% of the pixels were set to zero values to emulate the sensing by a CIS. The sparsified experimental coded image was processed by the TwIST algorithm and was decoded into a reconstructed image of the subject. The reconstructed images are shown in the right column of the figure. As a result of a visual assessment, the images of the subject were well reconstructed. The result validates the principle of the proposed imaging method in a real situation. The effects of using a real sparsified image sensor will be analyzed in future work.

Compared to simulations, there are three major differences in the conditions for imaging. At first, the chief-ray angle (CRA) characteristics of photo-detectors were restricted because we used a consumer image sensor designed for lens-based imaging. This fact resulted in vignetting, as mentioned in Ref. [6]. This minor problem can be solved just by the appropriate design of the image sensor for lensless imaging. Next, the PSF generated by physical phenomena in the experiment was not strictly space-invariant, whereas it was assumed as space-invariant in simulations. On the other hand, during image reconstruction, we approximated it as space-invariant also in the experiment in order to compute the forward model and its transpose quickly by using the convolution operation like Ref. [10]. This gap between the assumption and physics resulted in degradation of final image quality. If the matrical image reconstruction is adopted, this problem can be solved, though the computational cost significantly increases. In addition, in order to make a square image from rectangular captured data for fast image decoding, we padded zero values at the top and bottom area of the captured data. This caused discontinuity of the pixel value in a coded image. This affected the line-shape noises in the experimental reconstructed image. This can be solved by, e.g., improving the reconstruction algorithm exploiting the prior knowledge on discontinuous border in an image. In the experiments in this section, we repeated the same decoding processing three times independently, once for each color channel. However, aggressive utilization of the correlation of color-domain image information is a reasonable approach for improving the final image quality, as done in a computational compound-eye camera [21]. We will develop a decoding algorithm that exploits color-channel correlation in future work.

## 5. Discussion

The merit of the proposed lensless camera is the capability of super-FOV imaging with a thin and compact optical hardware constructed only by image sensors. The theoretical limit of the FOV for a single directional imaging is up to 180 degrees, which is the as same as that for a pinhole camera. However, the practical limit of the single FOV is restricted by the CRA characteristics of the detectors like the experiments in this paper. This problem can be solved by appropriate control of the CRA characteristics of the detectors in the manufacturing process. The FOV is also limited by the thickness of air holes mounted on the CIS. This limit can be solved by using more than two CISs with constructing regular-polyhedron-shape optical hardware, though the discussion in this paper focused on the use of only two CISs for theoretical simplicity. For example, the use of six CISs with the construction of a cube-shaped optical hardware is a promising approach to realize practical omnidirectional imaging. If the CMOS image sensor can be implemented spherically like in Ref. [22], a single CIS is sufficient to realize the omnidirectional lensless imaging, which should be the smallest and the simplest realization.

To realize the proposed camera, the physical implementation of the CMOS CIS is necessary. In future works, such a CMOS image sensor will be physically implemented, and the practical issues of using such a sensor will be addressed. The drilling of pixels on a CMOS image sensor itself was technically possible and demonstrated in past studies [23,24]. We regard that the same process can be used for implementing the proposed lensless camera. Afterward, the super-FOV imaging with the physically-implemented CISs will be demonstrated. The methods of optical encoding and computational decoding will also be improved by considering color-domain image correlation, aggressive design of the coding patterns, and so on. In the simulations, we used relatively high SNR values to clarify the effect of the air-hole ratio. However, in practice, there are situations where the SNR in captured data should be lower. A discussion on the noise robustness considering practical imaging situations will be addressed in a future work.

The proposed camera can realize both compactness and wide-FOV, which can be applied to rich information sensing from severely-limited space such as endoscopy, inspection, robot vision, wearable sensing, and so on. The implication of the proposed novel camera ranges from the improvement of the throughput of existing imagers to the pioneering new vision-technology applications.

## 6. Conclusions

In this paper, we proposed a novel architecture for a lensless camera that can capture the front and back scene at once. The merit of the proposed camera is the capability of super-FOV imaging by a thin and compact optical hardware constructed only by image sensors. To realize this, we exploited the CIS, which is the CMOS image sensor, whose pixels are randomly drilled into air holes. In the proposed camera, CISs are placed facing each other, where the front sensor works as a coded aperture and the back one works as a sparse image sampler of the coded optical image. The captured sparse coded image was computationally decoded into the subject image by the CS-based image-reconstruction algorithm.

We verified the proposed imaging system by simulations with numerical data and experiments with a real mask-based lensless camera and simulated air holes. The simulation and experimental results in this paper indicate the feasibility of the proposed lensless camera working with the sparse-sampling coded imaging. This indicates that the super-FOV imaging by a thin lensless camera, where both the front and back scene can be captured at once, is possible using the proposed architecture of Figure 1b if the CIS is physically implemented. The demonstration of the proposed super-FOV lensless camera using physically-implemented CMOS CIS will be a future work.

## Figures and Tables

**Table 1:** Specifications for the simulation.

Parameter	Value
Pixel count of object and reconstructed image	384×384 (pixels)
Pixel count of original image sensor (before drilling)	384×384 (pixels)
Pixel pitch in image sensor	10 (μm)
Size of cell in image sensor	60 (μm) × 60 (μm)
Size of elemental hole in drilled cell	40 (μm) × 40 (μm)
Thickness of each sensor device	1.0 (mm)
Distance between sensors	2.0 (mm)
Bit depth of signal from pixel	12 (bit)
Wavelength of light	532 (nm)

## Abbreviations

The following abbreviations are used in this manuscript:

CIS	coded image sensor
CMOS	complementary metal-oxide-semiconductor
FOV	field-of-view
CS	compressive sensing
PSF	point-spread function
TV	total variation
FWHM	full-width half-maximum
SNR	signal-to-noise ratio
PSNR	peak SNR
LCD	liquid-crystal display
CRA	chief-ray angle
